# The Hotspot for (Global) One Health in Primary Food Production: Aflatoxin M1 in Dairy Products

**DOI:** 10.3389/fpubh.2016.00294

**Published:** 2017-02-02

**Authors:** Chiara Frazzoli, Paola Gherardi, Navneet Saxena, Giancarlo Belluzzi, Alberto Mantovani

**Affiliations:** ^1^Istituto Superiore di Sanità, Rome, Italy; ^2^Local Health Unit, Piacenza, Italy; ^3^Central Institute for Research on Buffalo, Hisar, India; ^4^Ministero della Salute, Rome, Italy

**Keywords:** toxicology, risk assessment, risk management, climatic change, food security, food safety, India, Italy

## Abstract

One Health involves the multifaceted environment-animal-human web: nevertheless, the role of toxicological issues has yet to be fully explored in this context. Aflatoxin B1 (AFB1) contamination of feeds is a risk for the health of several farm animals, including fishes; milk is the only food of animal origin where a significant feed-food carry over may occur. The main AFB1-related compound present in milk is the hydroxy-metabolite aflatoxin M1 (AFM1). Besides contamination of raw milk, AFM1 is of concern for the whole dairy chain; AFM1 may also contaminate the milk of several other ruminants used for milk/dairy production. In a One Health perspective, milk represents a sentinel matrix for AFB1 vulnerability of the agro-food system, that is crucial in a phase when food/nutritional security becomes a global issue and climatic changes may affect agricultural productions. In the global setting, food chain exposure to long-term toxicants, such as AFM1, is a growing concern for economically developing countries, whereas global trade and climatic change makes AFM1 an emerging hot issue in economically developed countries as well. We critically review the state of the art on AFM1 risk assessment and risk management using two scenarios as case studies: a European Union country where the health system aims at ensuring a high-level protection of food chain (Italy) and the world’s largest (and economically developing) producer of dairy products by volume (India). The case studies are used to provide building blocks for a global One Health framework.

## Introduction

Aflatoxin B1 (AFB1) is a major toxic contaminant of foods and feeds; it is secondary metabolite of several *Aspergillus* spp. fungi affecting many food ingredients and feed materials, especially nuts (e.g., peanuts) and grains (mainly corn). *Aspergillus* molds can also concurrently produce other, less toxic AF metabolites (B2, G1, and G2). AF-producing *Aspergillus* spp. behave differently: *Aspergillus parasiticus* is more adapted to a soil environment, whereas *Aspergillus flavus* is more adapted to the aerial parts of plants. Contamination from *Aspergillus* may arise both in the field, as stressed plants may become infected, and/or during storage and transport (1). In the past, AFB1 contamination was thought to be mainly a problem of economically developing countries; in the last decade, climate changes have brought suddenly the attention to an enhanced risk in industrialized countries too, including Europe (2).

AFB1 is a potent hepatotoxicant and liver carcinogen; since it acts through a genotoxic mechanism, a tolerable daily intake cannot be set and human exposure should be reduced to levels as low as achievable. Tolerable levels (in the range from micrograms to nanograms per kilogram) have been set in Europe in various susceptible plant-derived food commodities as well as feed materials and complete feeds, based on the calculations of margins of exposure (1, 3). With regard to food-producing animals, AFB1 contamination of feeds is a risk for the health of several farm animals, including fishes; however, milk is the only food of animal origin where a significant feed-food carry over may occur (1). Thus, in a One Health perspective, milk may also represent a sentinel matrix for AFB1 vulnerability of the agro-food system, which may be crucial in a phase when food/nutritional security becomes a global issue and climatic changes may affect agricultural productions.

The main AFB1-related compound present in milk is the hydroxy-metabolite aflatoxin M1 (AFM1). Albeit less potent than AFB1, AFM1 presents similar toxicological hazards: in Europe, maximum levels for AFM1 have been set for consumable milk (0.05 µg/kg) and infant formulae (0.025 µg/kg) as parameters to reduce human exposure to the minimum, reasonably achievable level. Besides contamination of raw milk, AFM1 is of concern for the whole dairy chain, as it may be carried out to dairy products (4). Upon intake of contaminated feedingstuffs. AFM1 is also present in the milk of other ruminants used for milk/dairy production, such as water buffalo, camel, sheep, and goat (5). Since most of the available evidence deals with cow’s milk, AFM1 should be considered as a concern for all dairy productions.

## Global Aspects

### Global Trade and Food Security

In 1996, the Food and Agriculture Organization (FAO) stated that food security is set in “when all people, at all times, have physical and economic access to sufficient, safe and nutritious food to meet their dietary needs and food preferences for an active and healthy life.” Therefore, food safety is an essential part of food security. With global trade and climatic changes, food safety has emerged as a hot issue whose problems and solutions are transnational.

The global market has made AF contamination of feeds and milk in emerging countries a relevant problem in the industrialized world too. Already in 1989–1990, a UK survey on feed materials revealed high AFB1 levels in a number of samples imported from India, other parts of Asia and South America (6): ingredients at higher risk included those derived from sunflower, corn, and other oily seeds and cereals (7, 8). Whereas feed materials based on seeds, nuts, and grains draw most of the attention, the international trade of dairy products is a vulnerable segment as well.

India is the world’s largest producer of dairy products by volume, accounting for more than 16% of world’s total milk production, and it also has the world’s largest dairy animal population (9). Cattle and water buffalo milk are both major comparts of the Indian dairy sector, different from other dairy producing countries. The Indian dairy system is a low input–low output one; the average individual producer owns less than five cattle or water buffaloes and uses locally available feeds. This results in milk yields far below international averages and also in the world’s lowest production costs. In the 1990s, imports (0.4%) and exports (0.3%) were almost equal, but from 2001, India became a net exporter of dairy products (10). In 2010, the government and the National Dairy Development Board have drawn up a National Dairy Plan that intends to nearly double India’s milk production by 2020.^1^ In India, about 70% of the population lives in rural areas and about 38% of them are poor. For these people, as well as for the large vegetarian segment of the Indian population, dairy products provide a critical source of calories and animal proteins; per capita mean consumption of milk has been estimated at about 250 g per day, corresponding to more than 90 kg per year.^2^ Milk is consumed as whole milk by the majority of the Indian consumers, including infants and children, and liquid milk is a major component of the diet of Indian children.

In Italy, milk production in 2012 has been 10,876,191 t in the bovine sector, 192,000 t in the buffalo, 406,000 t in the sheep and 28,000 in the goat sectors (*CLAL, Dairy Economic Consulting firm*).^3^ The production trend is still largely seasonal, with a peak level in March–May. The area with the highest milk production is the Po valley in northern Italy, featuring among the main intensive agricultural areas in Europe, and in particular the region of Lombardy. The production system based on milk quota has characterized the milk sector in Italy since 1984, when the European Union adopted the quota system, up to 2015. The quota system has induced in Italy a steady production in the last 20 years and has prevented the milk price level to increase, thus forcing the farmers to keep under control the production costs and the supplies of raw materials for feed production. Milk production in Italy is undergoing a serious crisis due in large part to lower costs in other EU countries, so the national dairy industry increasingly relies on imports. To cope with the crisis, high-quality products, such as many *made in Italy* cheeses, are strategic because, despite higher costs, they meet high demand from international markets. Mean individual consumption of dairy products in Italy is calculated in 55 L of milk, 22.6 kg of cheese, 9.3 kg of yogurt and fermented milk, and 2.3 kg of butter per year in 2012 (*CLAL, Dairy Economic Consulting firm*, see text footnote 3). Further to “quality” products, “traditional” Italian products (i.e., products whose methods of processing, storage, and ripening have been consolidated over time, at least for 25 years) may run into the international trade, whereas “typical” Italian products are allowed for marketing in the production site only (Reg. 1151/2012, November 21, 2012).

In general, safety and security of milk and dairy products directly impact on public health and socio-economic development. It should also be considered that several opinions of the European Food Safety Authority (EFSA) on feed additives (11) and contaminants (12) pointed out that infants and children have higher intakes of dairy foods compared to adults, hence, are more exposed to substances present in milk. Among dairy products, prevention and management of AFM1 contamination of milk is a priority issue due to potential concerns for consumer’s health.

### Food Safety: State of the Art on AFM1 Risk Assessment

Risk assessment in food safety is defined for all populations groups, with a special attention for those identified as potentially more vulnerable. The One Health international use of terminology for risk assessment is driven by three standard-setting organizations, the Codex Alimentarius Commission (CAC) in relation to food safety, the World Organization for Animal Health (OIE) for animal health and the International Plant Protection Convention (IPPC) for plant health, under the Agreement on the Application of Sanitary and Phytosanitary Measures (SPS Agreement) of the World Trade Organization of which the European Union is a member. Regulation (EC) 178/2002, which establishes EFSA, contains definitions of a number of risk-related general terms which are similar to those provided by CAC. Although the European legislator does not dictate which of the three methodologies (and associated terminology) has to be used, should the major purpose of risk assessment be the regulation of international trade, the EFSA Scientific Committee concluded that particular care must be taken that the principles of CAC, OIE, or IPPC are followed strictly. EFSA Scientific Panels should identify which specific approach is most useful in dealing with their individual mandates, recognizing that different risk analysis standards have an impact on the terminology used by different EFSA Scientific Panels (13). Of course, in their turn, EFSA activities may (and should) contribute significantly to the development and updating of the scientific basis underlying OIE, IPPC, and especially CAC standards.

The characterization of a toxicological hazard in the food chain starts from the identification of health effects and of groups that may have an enhanced biological susceptibility, as well as the relationship between the extent and severity of effects and the intake level. In parallel, exposure assessment should consider the extent of exposure, as well as the most vulnerable food commodities and the most exposed population group(s), which may not be the same as the biologically susceptible group(s). Accurate, comprehensive, and comparable data on food consumption are crucial to assess risks.

#### AFM1 in Milk: Considerations on Toxicology and Carry Over

In ruminants, a considerable part of the ingested AFB1 is degraded in the rumen and does not reach systemic circulation. The absorbed fraction of AFB1 is transformed in the liver into a number of metabolites, including the hydroxy-metabolites AFM1, AFM2 (the analogous metabolite of AFB2) and AFM4. All AFM are excreted with milk, but AFM2 and AFM4 occur in milk at much lower concentrations than AFM1, thus are not considered as priority issues *per se*. AFM1 is a major AFB1 metabolite: it enters the systemic circulation or is conjugated in liver to glucuronic acid and excreted via bile: in its turn, circulating AFM1 can be excreted via the kidneys or be carried into milk.

Overall, AFM1 toxicological hazards, in particular hepatotoxicity and hepatocarcinogenicity (including genotoxicity), are comparable to those of the parent compound, even though AFM1 has a lower carcinogenic potency compared to AFB1, i.e., one or two orders of magnitude in experimental studies (14): considering that AFB1 ranks among the most potent carcinogens, AFM1 still retains a carcinogenic potential that is definitely worth of concern.

AF toxicosis in dairy animals does not represent a reliable alert for AF exposure and carry over into milk. Indeed, ruminants are generally less sensitive compared to non-ruminants because AFs are partly degraded by the forestomach flora. Most clinical signs recall liver dysfunction, such as anorexia, icterus, hemorrhages, and ascitis; at necropsy, the liver centrilobular necrosis and bile duct proliferation together with kidney lesions are fairly characteristic. In cattle, clinical signs occur after exposure to concentrations of 1.5–2.2 mg/kg feed, and in small ruminants even after exposure to >50 mg/kg feed. Early alerts might be represented by reduced milk production, photosensitization and, most important, reduced immune response including reduced response to vaccination. For such subtle effects, it is difficult to set a no-effect level: however, there is a margin of safety of at least 75 between toxic exposure levels (≥1.5 mg/kg feed) and the statutory limit (0.020 mg/kg feed) in Europe, which likely affords adequate protection (15, 16).

The excreted amount of AFM1 in the milk of dairy cows may represent at least 1–2% of the ingested AFB1; however, it is modulated by several factors (17). High-yielding dairy cows may show a higher carry over rate of AFM1 into milk, even above 6% of the ingested AFB1 (18).

Model calculations in Europe show that vulnerable high-yield cows exposed to feed with the current European maximum levels for AFB1 might produce in some cases milk with AFM1 levels above the European limit (19): the consumers of milk or dairy products from intensive, high-yield farming might be more exposed to AFM1, thus corroborating the magnitude of the AF problem both in low-scale and intensive farming. An important feature of AFM1 is the binding with the protein fraction of milk, and in particular the preferential binding to casein during milk coagulation (20). Therefore, AFM1 is liable to concentrate in cheese and other dairy products with a high protein content. Finally, there is widespread evidence of AFM1 carry over into the milk of other ruminant species (5, 20), but a thorough framework to assess the species-specific kinetics is lacking.

#### Is Aflatoxicol an Issue?

Aflatoxicol in a main metabolite of AFB1 in many species, from humans (21) to salmonids (22). Aflatoxicol has been somewhat overlooked, as it is even not mentioned in the EFSA opinion on aflatoxins in feeds (1); however, this metabolite is suspected to be an endogenous reservoir of AFB1 in the organism. Indeed, in poultry, aflatoxicol is the main component of total AF residues, with highest content in liver (23).

In ruminants, the situation may be different: in calf liver preparations *in vitro*, M1 and Q1 were the major chloroform soluble AF metabolites, with small amounts of aflatoxicol (22). In two cows given a single oral high dose (0.5 mg/kg_bw_) of AFB1, aflatoxicol was just a minor component of AF residues in cow’s milk: the ratio of the concentrations for aflatoxicol, AFB1 and AFM1 was approximately 1:10:100, respectively (24). Also in the milk of goats experimentally treated with AFB1, aflatoxicol was present in trace amounts only whereas AFM1 predominated (25).

This data are not in accordance with an extensive study carried out on pasteurized cow’s milk marketed in Mexico. Aflatoxicol was detectable (≥0.05 μg/L) in 13% of samples, 8% showing levels ≥0.5 μg/L: the upper value was 12.4 µg/L. AFB1 was present mainly in traces, the highest value being 0.4 μg/L. Autumn samples were significantly more contaminated with aflatoxicol, while no relationship was found with milk fat content (26). Interestingly, the same Mexican survey found that aflatoxicol concentrations were overall of the same magnitude order as those of AFM1 (40% of samples ≥0.05 µg/L, 10% of the samples ≥0.5 µg/L, upper value 8.35 µg/L) (27).

In real-life situations, exposure to contaminated feed may be a prolonged, low-level one or may follow a repeated pulse-like pattern: it might be possible that these scenarios would result in different metabolism of AFB1 compared to findings of the limited experimental studies, using high-dose short-term exposures. On a practical ground, and pending more robust data, one cannot rule out altogether that aflatoxicol might be monitored in milk and dairy products concurrently with AFM1 in order to achieve a sound estimation of consumer’s exposure.

Interestingly, an isolated paper reported that aflatoxicol may bind to bovine uterine estrogen receptors *in vitro*, although its potency is much lower than the strong estrogen-agonist mycotoxin, zearalenone (28): to our best knowledge, the role of aflatoxicol as endocrine disrupter in the disorders of reproduction or lactation of cattle has not been further explored, nor any possible significance for consumers safety.

### Traslational Research: State of the Art on AFM1 Risk Management

The FAO states that *the primary goal of the management of risks associated with food is to protect public health by controlling such risks as effectively as possible through the selection and implementation of appropriate measures* (29). The overall objective is to undertake legitimate measures to protect the health of consumers (in relation to food safety matters) at a level they consider necessary (sometimes defined “protection goals”) in a consistent and transparent way while prohibiting unjustified restrictions of trade; thus, risk management should encompass proportionate, targeted, and effective measures.

The established prevention strategy of AFM1 contamination of milk is mainly good practice along the feed production chain, including the primary production of feed ingredients. In fact, aerobic in nature, mycotoxic fungi need air, moisture, nutrients, and suitable temperature for their growth and metabolism.

Climatic conditions in India are most conducive for mold invasion, proliferation, and production of mycotoxins. The high-risk areas in India are Kerala, Western India, Gangetic plains, north eastern and coastal areas of Andhra Pradesh, Karnataka, and Tamil Nadu. Unseasonal rains and related flash floods are very common in India, and this enhances the moisture content of the grains and therefore its vulnerability to fungal attack (30).

The high-risk area in Italy is the Po valley that is at the same time also the most milk productive area and the area whose climatic gradient is at highest risk. The average humidity rate here is about 80%; Piacenza, a town located in the center of the valley, shows an annual average of 80.1%. Apart from climate, climate changes (i.e., aspects like changes in temperature, relative humidity, insect attack, drought, and stress condition of the plants) influence the ability of molds to produce mycotoxins (2).

Due to the worldwide recognized problems expected for food and feed safety in relation to climatic changes, AFs in cereal crops can be listed among emerging risks. The EFSA Scientific Committee in 2007 stated that “*an emerging risk to human, animal and or plant health is understood as a risk resulting from a newly identified hazard to which significant exposure may occur or from unexpected new or increased significant exposure and/or susceptibility to a known hazard*” (31). Thus, AFM1 in milk is a well-known risk which, due to changing scenarios, shows an increasing and still poorly predictable exposure pattern. The emerging risks identification requires a high level of expertise due to the data gaps and uncertainties in the evaluation process. Since 2010, EFSA has provided scientific criteria and recommendations to address consistent and up-to-date activities on emerging risks in Europe and European Member States; since 2012 a Standing Working on Emerging Risks is on place (32). In Italy, the National Reference Centre on Emerging Risks has been implemented in Milano (Lombardia Region) as a structure of the Istituto Zooprofilattico of Lombardia and Emilia (located in Brescia): currently, main activities concern procedures and methodologies to assess and collect data sources and reinforcement of a knowledge exchange network inside and outside Italy, involving other institutions and stakeholders in conformity with the Regulation CE 178/2002 (33). The Italian system is definitely in place for biological hazards and animal diseases; other aspects, including emerging toxicological hazards, deserve implementation and strengthening of the expertise network.

#### Prevention in the Dairy Chain: Manageable Aspects

Control of AFM1 is routinely practiced in many industrialized and emerging countries, but the cost to track contamination continuously is hardly sustainable. No doubt, a consistent net of controls performed according to validated methods provides a highly valuable support both to reducing consumer’s exposure and mainly to monitoring the space and time trends; however, stand-alone controls would present a remarkable shortcoming. Rejection of milk as unfit for consumption, hence food wastage, would be the only possible solution, especially when a significant sample fraction exceeds a given regulatory limit. Therefore, controls should be intended as the downstream component of a prevention strategy aimed at reducing consumers’ exposure, primarily through the prevention of AFM1 contamination. AF contamination of crops in the field is the most critical step in Europe. Apart from weather conditions, the following points impacting on the quality of raw feed ingredients represent the main known manageable factors contributing to the occurrence of AF in milk.

##### Feed Chain Facilities

Since the 1990s, an increase attention toward AFB1 contamination in Mediterranean Europe revealed that corn silage is a vulnerable item: during ensiling, under unfavorable circumstances, high temperature can facilitate the growth of toxigenic *Aspergillus* spp. Here is a list of critical factors (34, 35):

- Soil contamination by *Aspergillus* spores may be increased by modern cultivation systems excluding crop rotation, frequent irrigations with fixed modern equipment, and leaving a presence of infested and damaged pods in the field.
- Cultivar selection that disregard vulnerability to *Aspergillus* spp. as a selection criterion.
- Moisture content of grain or relative humidity surrounding the substrate.
- Delay in harvesting corn.
- Breaking of grains due to threshing machines or insect/rodent attack, implying an increased presence of impurities and grain fragments.
- Poor storage conditions, in particular when grains are stored without artificial drying phase in the wet seasons.
- Transport conditions, when grains are loaded and/or transported in wet and closely packed conditions (lack of aeration).

Further to ethic and scientific responsibility, the legal aspects linked to the EC Regulation no. 178/2002 (33) require that feed business operator implement a traceability system for the identification of corn stocks.

The corn suspected of contamination should be clearly identified, stored in separate compartments of the premises that should be easily distinguished from those containing the safe product. The level of AFs contamination should be considered during the pre-marketing phase to make choices based on the results of self-monitoring: the different batches will be sold as human food ingredient, animal feed material, or other (e.g., industrial) purposes. Corn having AF levels greater than the maximum legally tolerated levels must be destroyed: also industrial usages are not allowed (33). The current EU legislation does not allow dilution of corn or other feed material batches with AF levels above the legal limits at feed factory level. The European approach considers that not allowing dilution is a powerful mean to stimulate all operators throughout the chain to apply the necessary prevention measures to avoid contamination as much as possible. Last but not least, the same approach applied to feedingstuffs for dairy cows must be applied to feedingstuffs for small dairy ruminants.

##### Farm

In both cases of feed manufacturing in-house and feed purchasing, the farmer should pay special attention to the preliminary check of corn stocks in order to verify safety through standardized sampling procedures.

The experienced check of quality and origin of feed materials at farm is all important, especially in economically developing countries, where most farmers do not have a consistent technological support. Clean livestock feed holds the key to clean milk. The majority of farmers in most milk-producing states in India feed cereals or agricultural/oilseed by-products to their dairy animals. Such AF-vulnerable feed materials as cereals (maize, sorghum, etc.) and oilseeds (peanuts, soybean, etc.) constitute more than 70% of cattle feed (30). Moreover, the food that is declared unfit for human consumption often finds its way as feed for animals. Indeed, a number of reports indicate the presence of high concentrations of AFs in cattle feed in India; the situation may be worsened by the adoption of new techniques for feed preserving without due considerations for safety, e.g., silages are more vulnerable to *Aspergillus* if anaerobic conditions are not strictly controlled (36).

##### Strategies to Minimize Feed Contamination by AFB1

Clean livestock feed holds the key to clean milk. Intervention practices point at reducing AF contamination in the field and preventing AF formation during storage. New techniques for preserving green fodder such as silages are unsafe if anaerobic conditions are not strictly controlled (e.g., artificial drying in the whet seasons).

###### Selection of Resistant Cultivars

Strengths and weaknesses of biological control (e.g., breeding for introduction of a atoxigenic strain to the crop environment to compete with toxigenic strain) and enhanced plant resistance (e.g., resistance to the fungus, inhibition of AF biosynthesis, resistance to insects) have been reviewed, as well as relevant challenges in economically developing areas (34, 35).

###### Silage Additives

Worldwide, a high proportion of the ruminant diet consists of silages made of forage crops. In practice, silages are often contaminated with mycotoxins, including AFB1: when silage conditions are inadequate, a significant production of toxins may occur also during ensiling. In the large mass of ensiled feed, mycotoxin may be not distributed homogenously, rather, it may occur in some hot spots. Several feed additives, either chemicals or bacterial strains, are proposed to improve the ensiling process in Europe. Thus, it is relevant to know their effect, if any, on AF production and persistence. The use of formic acid appeared to somewhat favor the production of AFB1 and is discouraged in Europe (37); conversely, interventions with microbial additives that can enhance aflatoxin degradation can be a promising strategy (38).

###### Feed Additives

Mycotoxin binders/adsorbing agents to *reduce AF bioavailability* are permitted only in complete feeds with levels of AF or other mycotoxins not higher than the maximum tolerated limit. Indeed, the EFSA has a quite strict approach toward feed additives intended as mycotoxin binders. Several compounds successfully reduce the bioaccessibility of AFs from contaminated feeds *in vitro*. The treatment of contaminated feeds with mycotoxin binding agents may be useful to protect animal health and avoid milk contamination by the carcinogenic AFM1 metabolite. However, mycotoxin binders may impact animal health, e.g., by interfering with the absorption of nutrients or medications (39). A potential alternative strategy is to act on the *Aspergillus* metabolism within feedingstuffs, by inhibiting AF biosynthesis or promoting degradation into non-toxic metabolites by biotransforming agents such as bacteria/fungi or enzymes (39). The EFSA approach toward feed additives intended to reduce AF contamination is consistent with the general European policy identifying a high level of food safety (40) and prevents unsafe material to be recovered for use in the food chain. On the other hand, one might argue that making no attempt to recover contaminated feeds would eventually lead to wastage of resources and to a weakening of dairy chain sustainability, especially in economically developing countries scenarios other than Europe. Local practices in developing countries may be investigated for their effectiveness: interestingly, lactic acid fermentation of grain-based materials may result in AF degradation (41). In all cases, considering the serious risks for consumer’s health related to AFM1 in milk, approaches to recover contaminated feedingstuffs should be strictly regulated and surveyed.

##### Strategies to Minimize AFM1 in Living Animals and Their Products

###### Animal Detoxification Systems

Once ruminants are exposed to AF, attempts may be made to support the animal’s capacity for detoxification either in rumen or liver.

###### Processed Dairy Products

In India, processing of milk is limited to pasteurization or fermentation, and both these methods are not capable of reducing AF or its metabolite. In fermented dairy products, AFB1 is transformed into the non-toxic AFB2 and the less toxic aflatoxicol (42). Although no information is provided on AFM1, this finding may indicate that transformation of milk into fermented products could be a strategy for risk reduction in areas with high AFM1 contamination.

#### Operational Aspects

##### European Scenario

Similar to the approach adopted in different contexts for other high-concern contaminants, like dioxins, Europe considers two official thresholds for AFM1 in milk, a *alert threshold level* calling for action (0.04 µg/kg) and a *maximum tolerated level* (0.05 µg/kg) (43). When the *alert threshold level* is exceeded, the business food operator must inform the competent authority (CA) within 12 h and propose the corrective measures to apply; in general, these refer to good farming practices, e.g., modification of animal diet by reducing or cutting the feed material/source having the highest risk of contamination. Thus, whereas dilution of contaminated feed is not accepted as a standard risk management practice at feed factory level, it can be accepted as a temporary measure in the farms where the threshold level in milk is exceeded.

When the *maximum tolerated level* is exceeded, the business food operator must inform within 12 h the CA and all other food chain operators that have been supplied with the contaminated milk. Provisions are then dictated by the EU regulation and include suspension of milk delivery and/or sale, starting procedures for withdrawal from the market, and elimination of contaminated milk (44). A key tool to ensure the cross-border follow of information is RASFF, the Rapid Alert System for Food and Feed. RASFF ensures that urgent notifications are sent, received, and responded to collectively and efficiently. Currently, in Europe, the self-monitoring plan must assure the compliance with the maximum tolerated level of AFM1 (43). To make the monitoring effective, at least one sample of milk should be taken twice a week; most important, the plan should take into consideration risk categorization parameters, namely, the territory (e.g., climatic conditions), the production volumes, the results of previous controls as well as additional risk factors like the modification of the daily feeding rate or the opening of a new corn silage. A reliable tracking system for feed materials, and also for purchased animals, is a necessary complement to the self-monitoring plan at dairy farm level. At the level of dairy factory, a monitoring plan should be established taking into the risk categorization parameters mentioned above in order to identify farms, farm clusters or farming areas calling for an enhanced level of attention. At dairy factory level, where milk is often collected from multiple and different sources, it is especially critical to have a robust tracking system in place.

Finally, since the global market requires co-ordination of control activities and an overall strategy for risk management, since 2007 the EFSA is building a framework for collection of national dietary survey data from European Member States.

##### The Indian Scenario

Constraints in controlling AFM1 contamination are currently a complex problem in the emerging Indian scenario. Millions of small dairy owners who produce more than 60% of India’s milk are resource-poor farmers with scant space and money for storing feeds and feed ingredients. The dairy industry that relies on milk supplies from such livestock owners needs to test samples for AF before pooling the milk for industrial processing; this may not be practical as testing and quantifying for each vendor is neither economical nor feasible. India has limited feed resources to meet the needs of a huge population of cattle and buffalo, while production of grains for direct human consumption has priority. This scarcity of feed resources forces the farmers and dairy owners to compromise on the safety and quality of feeds in order to fulfill the nutrient requirement of their livestock. Furthermore, these farmers, even though individually small and marginal, contribute altogether a major portion of milk to the dairy processing industries through milk unions/cooperatives; hence, traceability from such a multitude of rural enterprises remains a problem.

Several papers report data in AFM1 contamination of milk and dairy products in India (41); however, whereas many reports are issued, the reliability of findings and conclusions drawn is questionable. Several reasons do suggest caution. Sampling procedures may not be appropriate for ensuring true representation of contamination in the cattle population. Also, on many occasions, analytical methods used are either not appropriate or not properly validated so as to achieve desired accuracy. Further, these analyses may be done in non-accredited laboratories. There is a widespread recognition that a problem does exist, but the awareness on how to investigate it should be improved. However, recently the Food Safety and Standards Authority of India (FSSAI) laid down regulations/guidelines (45) on sampling and analytical procedures to be followed for different chemical contaminants in various commodities/feed ingredients/mixtures for surveillance purposes. A legal limit for AFM1 is established at 0.5 µg/kg; however, while industrialized countries have set maximum permissible limits for AF levels in livestock feed, no legal limits exist for livestock feeds and fodder in India. Indeed, feed, rather than downstream control of milk, is the key point for AFM1 risk management.

In general, economically developing countries may adopt the maximum tolerated levels of AFB1 in feeds or AFM1 in milk as Europe or other industrialized countries; however, risk management may be different. In particular, in situations where food security is less consolidated than in Europe, consideration may be given to minimize wastage of food with high nutritional value. Besides the use of mycotoxin binders in feeds to reduce uptake by animals, dilution of contaminated feedingstuffs seem to be the preventive action of choice. In the case of contaminated milk, to date no reliable procedure to decontaminate milk for human consumption, other than dilution, is available.

#### Regulatory Aspects: The Food Safety Assessment and Management Structure in the Frame of the European Hygiene Package and the Role of Self-monitoring

The current regulations about food safety in Europe (Hygiene Package, collecting Reg. CE 852, 853, 854, 882, 2004), following the principles of the European Strategy for Food Safety (2000), clearly distinguish responsibilities and roles: the food business operator is the primary responsible assigned to guarantee the safety of feed and food that is put on the market. The tool in charge of the food operator is primarily the self-monitoring plan that is approved by the CA, systematically updated along with any foods process modifications, and then confirmed by the same CA. The programing of official monitoring activities is aimed to check the application of self-monitoring by the food-producing enterprise. Consequently, it is important that the public services responsible for food safety make available consistent, updated and evidence-based tools in order to support and facilitate risk prioritization and management by enterprises.

The toxicological characteristics and potential exposure of the general population, including children, make AFM1 a priority issue for the dairy chains; accordingly, a specific program should be in place for monitoring of AFM1 on raw milk delivered at processing plants. Such program should indicate the frequency of sampling, which should be based on both the production capacity and on-risk categorization indicators; the method of analysis, which must have been accredited; the tracking system of every single supplier; the corrective measures to be taken in the event of alert or maximum tolerated levels being exceeded; last but not least, operational guidelines should also include management actions in case of higher risk situations, such as when environmental and climatic conditions can increase the levels of contamination in corn or other major feed materials (34).

The high rate of increased levels of AFB1 in corn and AFM1 in milk in Northern Italy in 2003, in relation with highly unfavorable climate conditions (high temperatures, drought, and strong insect damage), was efficiently managed through a food chain approach that significantly reduced the chance for consumer exposure. The event of 2003 pointed out critical phases of self-monitoring. In Italy, there have been several recent alarms on corn contamination with AF related to changing climate conditions and the consequent presence of AFM1 in milk: this situation has prompted the Ministry of Health to issue a contingency plan (i.e., extraordinary operative procedure for the prevention and risk management of aflatoxins contamination in the dairy chain and in the production of corn for human and animal consumption in extreme climatic condition) to deal with emergency situations that may jeopardize both consumer’s safety and the availability of nutrients from dairy products (46). The Italian Health system is highly characterized by One Health. It has two main characteristics. First, its remit includes all veterinary topics, including feeds, which is indeed rather unusual among EU member states. Besides reflecting the spearheading role of the Italian school in the development of veterinary public health, this approach has been adopted by European bodies (DG SANCO and EFSA) and it is consistent with the conceptual framework “from farm to fork.” Second, the structure of the Italian Health system (in particular the food safety system, including official control and risk assessment in food safety) is shaped like a broad-based pyramid; the Ministry of Health provides the general policy to the regions, which have a strong autonomy in allocating resources. More in detail, the pyramid is structured at three levels: the Ministry of Health (first level) is the central CA for risk management; for risk assessment, the Ministry is assisted by the National Health Institute (ISS) and by the National Food Safety Committee, an independent expert body hosted in the Ministry premises. Since the system is a federal one, policies relevant to the management framework in the territory have to be negotiated within the State-Regions Council, that deals with all matters when the central authority overlaps with the (21) regional autonomies (second level). The federal approach to health matters is in place since 10 years and is now under debate because of several negative instances, including inconsistent approaches and lengthy political negotiations hindering decision. Within regions, the system is broken up in (146) local health units (LHUs), that are in charge of managing the risk on the territory (third level). Each LHU has a Prevention Department that includes a Veterinary service, divided in three areas (Animal Health and Welfare, Food Safety and Hygiene of establishments and premises). The LHUs lists the farms according to risk categorization criteria and assess both the resources available and the needs for intervention. The Food Safety area of the veterinary service is the territorial body in charge of both carrying out the official control in food safety and adopting suitable measures and actions for risk management, which include quantification of costs and reimbursements, if due.

The effectiveness of the official control system is continuously monitored through a randomized or targeted comparison with the self-monitoring system, which, to date, is based on farm’s management documents and analytical data produced by the 10 Istituti Zooprofilattici (Institutes for the animals health and safety of food products). Such comparison is usually mainly based on sample monitoring by the LHU system and its annual distribution of resources that must, of course, take into account also other items (compliance with international plans, audits). Last but not least, the European Commission developed, since the early 1990s, a hierarchical network of Community Reference Laboratories (CRLs) and National Reference Laboratory (NRL) in the Member States (47). This CRL-NRLs system aims at controlling and coordinating the work carried out by routine field laboratories commonly entrusted with analysis of residues and contaminants in Europe. The Institute for Reference Materials and Measurements of the European Commission Joint Research Centre is the CRL for AF, including AFM1, whereas the NRL is located at the Italian National Health Institute.

### What Can Be Done More

#### India: What Can Be Done in the Frame of the Food Safety and Standards Act

In India, food safety has been recognized as an important component in protecting the health of people. However, in view of widespread poverty and malnutrition in economically developing countries like India, programs directed toward food security (to satisfy caloric needs and minimize hunger and malnutrition) have precedence over programs designed to ensure wholesomeness, quality, and safety of food.

In order to meet the global standards, the Government of India enacted an integrated food law called the *Food Safety and Standards Act* in August 2006, which came into effect from August 2011. The new FSSAI, established under this Act, has consolidated various policies setting the requirements for food safety, including machinery, premises, quality control, certification, packing, marking, and labeling standards for all food products; the Act aims at regulating food safety in India through one overarching regulation. Maximum tolerated levels for both domestically produced and imported milk and dairy products have been set by the authority for most of the contaminants and toxicants. The permissible limit for AFM1 in milk and dairy products is 0.5 µg/kg prescribed by the mandatory regulations of the country (FSSAI: Food Safety and Standards Rules 2011), in accordance with the CAC. As dairy product prices and income of dairy production continue to increase, the average dairy herd size is also increasing. In addition, interests from corporate investors have also facilitated construction of larger dairies partnering with dairy processors. Thus, Indian scenario is changing, and food safety standard and tools should cope with such change.

#### Integrating Biomarkers into the Control System

The European strategy for food safety (40) empowers the risk assessment approach and the “from farm to fork” principle. In the new EC perspective, the Official control must be increasingly integrated by renewed systems for self-monitoring by food business operators.

The ethical, scientific, and legal responsibility of food operator in the safety and quality of food products they put on the market requires the definition of good practices, self-monitoring plans (including Hazard-Analysis and Critical Control Points, or HACCP, of course) and traceability systems. On the other side, self-control plans like the mentioned two analyses per week have the weakness of being carried out basing only on statistical and economical criteria. Innovation in the food chain requires the optimization of results obtained from the resources devoted to self-control activities. In this view, the drivers for decision-making in self-control plans should be increasingly based on scientific inputs rather than statistics only.

On its side, scientific research is called to develop cost- and time-effective field methods/tools that can be transferred for self-control purposes. Innovative methods are also expected to complement the consolidated European system for official control: this is based on sophisticated and expensive laboratory instruments and techniques that require extensive sample pre-treatment and personnel training, e.g., multi-analytic method based on liquid chromatography-electrospray ionization tandem mass spectrometry (48). Moreover, costly analytical methods imply that the sample is transferred from the field to the laboratory. This approach needs integration by validated biomarkers that can be increasingly emerging as measurable biochemical or molecular (parent toxin itself) indicators of contamination. They should be monitored directly on the farm or dairy factory to screen daily production and eventually allow timely corrective action. These biomarkers should be transferable, i.e., validated by the establishment of a dose–response relationship, and reliably measured under conditions of use and by food business operators. Biomarkers should be sampled in living animals; thus, matrices are blood/serum, milk, urine, feces. AFM1 in milk is a direct and relevant biomarker of exposure of AF in ruminants; further research is needed to identify biomarkers of effective dose, i.e., indicating that concentrations of AFM1 are reaching levels that may have relevant biological activities.

The biomarkers approach should be developed to complement the consolidated European system for official control (based on sophisticate laboratory instruments), thus implementing an integrated top-down and bottom-up approach (49, 50). This is particularly important for primary productions in economically developing countries, where environmental conditions and poor resources stress both chances of contaminations and challenges for prevention (51).

Promising technologies are being developed to prevent (e.g., heat, humidity, and antioxidant power of the environment) and early detect fungal contamination and remove materials containing fungi: tools include tests for chemical or physical changes occurring with fungal growth like electronic noses and tongues. Among possible field tools, biosensors for AFB1 are based on indirect assays, i.e., the presence of the AF is established by its interaction with a biological medium immobilized on the surface of the probe, either an antibody that selectively binds the antigenic AF (immunosensor) or an engineered micro-organism (bioluminescent whole-cell biosensor). Recently, proposed sensors are based upon the inhibition of enzymes. The biochemical (binding or inhibition) event triggers a signal that can be detected by its optical, acoustic, or electrochemical properties: the advantages of electrochemical assays may include the low cost of production of the electrodes, amenability to miniaturization, and multiplexing (52). Critical points during development of field methods are matrix effects and use conditions (farm is not a university laboratory) as well as the need for a time-effective sample preparation, as well as measurement. Mammalian cell-based biosensors may detect active concentrations of toxic substances and are promising for field application due to their high speed, low cost, and considerable sensitivity (53). Some early metabolic effects might be useful to develop biomarkers of effective dose. AFM1 impairs the mitotic process, without effects on cell viability (54). AFB1 in rats has been associated with hypocalcemia, a decrease in absorption of calcium, and the impairment of availability of bile salts; the mechanism was the decrease of Vitamin D3 production and lipid absorption, which might be early effects at intestinal and/or feed conversion level. Additionally, AF affect also the bioavailability of other essential minerals including iron, phosphorus, and copper (55). Effects on these essential minerals would likely be related to reduced antioxidant response and also reduced immune response (e.g., impaired immunoglobulin production), which have both been related to AF exposure in farm animals. It would certainly be worthwhile to assess whether these early metabolic changes can be used as early biomarkers in milk, in order to support early intervention under self-monitoring practices (50).

Of course, no single metabolic parameter would have the appropriate specificity to signal a possible presence of an active concentration of AFM1; however, a panel of different parameters may be investigated as an AF “fingerprint.” Such approach requires the investigation of the dose–response relationship linking the intake of AFB1, the presence of AFM1 in milk, and the possible metabolic biomarkers. Analogously, co-occurrence of mycotoxins different from AFM1 in milk should be investigated (56).

#### Endorse Scientific Research

With regard to AF, the following research needs are highlighted:

– Selection of cultivars of maize and other relevant crops that have reduced susceptibility toward the fungal infestation. The maize, third worldwide crop, needs protection at the production level.
– Integrated prevention strategies at pre-harvest or postharvest times, including (when required and feasible and upon a risk-benefit analysis) the search for methods of mycotoxin decontamination.
– Field study to assess prevention strategies in the field (including cultivar selection) as well as in feedingstuffs. Applicability (field studies) of prevention methods should be verified in the presence of climatic and pedoclimatic conditions as well as different farming methods.
– Sensitive and cost-effective methods for detection and screening of AF (including aflatoxicol) in feed and milk exploiting immunochemistry and sensor/biosensor technology. (Bio)sensor arrays have the potential to become widely accepted as a system for early alert and self-monitoring applications, provided that robust results on fully automated platforms are successfully generated and grids of (bio)markers are validated. This will result in higher protection of animal and human health and enormous cost saving to food business operators through the prevention and reduction of product recalls and reduced treatment costs. Fabrication techniques of the microelectronics industry, microchemical sensors and biosensors, novel artificial receptors for recognition of specific mycotoxins in conjunction with, for example, microchemical sensors, offers novelty in both recognition and transduction process. Such tools offer a realistic route to the development of analytical measurement systems for the rapid, on-site (out-of-laboratory) assessment of food raw materials and processed food.
– Update of estimate model for AFM1 carry over in consideration of developments in production systems and animal nutrition and, most important, in all relevant milk-producing species. These considerations and the toxicological risks related to AFM1 call for prevention, rather than management upon a crisis onset, considering that there is clear evidence that also feed ingredients from advanced economies may expose to high levels of AFB1.
– Strategies for farmers’ information and risk perception to support the empowerment and proactive role of food primary producers in the protection of public health.
– Development of models for the prediction of biogeographical agricultural scenarios of cultivated plants as well as the related molds/mycotoxins.

## Conclusion

The detection of AFM1 in milk is the direct and most appropriate biomarker of internal dose to assess and measure whether a dairy animal is exposed to the toxicity of AFB1, as well as to assess and verify the efficacy of any corrective action. At the same time, the detection of AFM1 is also a biomarker of human dietary exposure to a toxic contaminant such as AFM1. Under this view, the possibility of daily management of AFM1 level through biomarkers is a challenge for both human and animal health, i.e., for the One Health framework. The project ALERT^4^ focuses on self-monitoring in the dairy chain. Indeed, milk is both highly consumed by infants, highly vulnerable to toxic contaminants, suited sentinel matrix for environmental monitoring purposes, and business core of a particularly precious and suffering group of food business operator like farmers. ALERT has the purpose of identifying and characterizing innovative metabolomic-based biomarkers for early warnings based on production and product anomalies and self-monitoring purposes, designing modern HACCP plans including tools to manage the toxicological risks, and establishing a long-term dialog between producers and research bodies for strengthening innovation (49). Regulatory (i.e., top-down) measures may have little impact in remote rural areas and in family farming communities in economically developing countries: here, bottom-up and communication activities are particularly crucial (49).

## Author Contributions

The authors contributed equally to the paper.

## Conflict of Interest Statement

The authors declare that the research was conducted in the absence of any commercial or financial relationships that could be construed as a potential conflict of interest.

## Footnotes

^1^http://www.nddb.org/information/stats/milkprodindia.

^2^http://www.nddb.coop/ndpi.

^3^http://www.clal.it.

^4^http://www.alert2015.it.

## References

[1] EFSA. Opinion of the scientific panel on contaminants in the food chain [CONTAM] related to Aflatoxin B1 as undesirable substance in animal feed. EFSA J (2004) 2(3):1–27. 10.2903/j.efsa.2004.39

[2] Miraglia M Marvin HJ Kleter GA Battilani P Brera C Coni E Climate change and food safety: an emerging issue with special focus on Europe. Food Chem Toxicol (2009) 47(5):1009–21. 10.1016/j.fct.2009.02.005 19353812

[3] EFSA. Opinion of the scientific panel on contaminants in the food chain [CONTAM] related to the potential increase of consumer health risk by a possible increase of the existing maximum levels for aflatoxins in almonds, hazelnuts and pistachios and derived products. EFSA J (2007) 5(3):1–127. 10.2903/j.efsa.2007.446.

[4] Santini A Raiola A Ferrantelli V Giangrosso G Macaluso A Bognanno M Aflatoxin M1 in raw, UHT milk and dairy products in Sicily (Italy). Food Addit Contam B (2013) 6(3):181–6. 10.1080/19393210.2013.780186 24779902

[5] Rahimi E Bonyadian M Rafei M Kazemeini HR Occurrence of aflatoxin M1 in raw milk of five dairy species in Ahvaz, Iran. Food Chem Toxicol (2010) 48(1):129–31. 10.1016/j.fct.2009.09.028 19786054

[6] Ministry of Agriculture, Fisheries and Food. Mycotoxins: Third Report. The 36th report of the Steering Group on Chemical Aspects of Food Surveillance. London, UK: HMSO (1993). p. 24–34.

[7] Blüthgen A Ubben EH Survey of the contamination of feeds and tank bulk milk with aflatoxins B1 and M1. Kieler Milchwirtschaftliche Forschungsberichte (2000) 52:335–54.

[8] Scudamore KA Hetmanski MT Chan HK Collins S. Occurrence of mycotoxins in raw ingredients used for animal feeding stuffs in the United Kingdom in 1992. Food Addit Contam (1997) 14:157–73. 10.1080/02652039709374511 9102349

[9] Singh R Indian Dairy and Products Annual 2010. USDA Foreign Agricultural Service. Global Agricultural Information Network (2011). Available from: https://gain.fas.usda.gov/Recent%20GAIN%20Publications/Dairy%20and%20Products%20Annual_New%20Delhi_India_12-23-2010.pdf

[10] IUF. Indian Dairy Industry. IUF Dairy Industry Research. (2011). Available from: http://www.cms.iuf.org/sites/cms.iuf.org/files/Indian%20Dairy%20Industry.pdf

[11] EFSA. Scientific opinion on the safety and efficacy of iodine compounds (E2) as feed additives for all animal species: calcium iodate anhydrous and potassium iodide, based on a dossier submitted by Ajay Europe SARL. EFSA J (2013) 11(2):3099 10.2903/j.efsa.2013.3099

[12] EFSA. Scientific opinion on polybrominated diphenyl ethers (PBDEs) in food. EFSA J (2011) 9(5):2156 10.2903/j.efsa.2011.2156

[13] EFSA. Scientific opinion on risk assessment terminology. EFSA J (2012) 10(5):2664 10.2903/j.efsa.2012.2664

[14] Henry SH Whitaker T Rabbani I Bowers J Park D Price W Aflatoxin M1. In: The Fifty-sixth meeting of the Joint FAO/WHO Expert Committee on Food Additives (JECFA) editor. Safety Evaluation of Certain Mycotoxins in Food. Rome, Italy: Food and Agriculture Organization of the United Nations (2001). p. 1–102.

[15] Miller DM Wilson DM Veterinary diseases related to aflatoxins. In: Eaton DL Groopman JD, editors. The Toxicology of Aflatoxins: Human Health, Veterinary and Agricultural Significance. NY: Academic Press (1994). p. 347–64.

[16] Shane SM Economic issues associated with aflatoxins. In: Eaton DL Groopman JD, editors. The Toxicology of Aflatoxins: Human Health, Veterinary and Agricultural Significance. New York: Academic Press (1994). p. 513–27.

[17] Van Egmond HP Aflatoxin M1: occurrence, toxicity, regulation. In: van Egmond HP, editor. Mycotoxins in Dairy Products. London; New York: Elsevier Applied Science (1989). pp. 11–55.

[18] Veldman A Meijst JAC Borggreve GJ Heeres-van der Tol JJ Carry-over of aflatoxin from cow’s food to milk. Anim Prod (1992) 55:163–8. 10.1017/S0003356100037417

[19] EFSA. Opinion of the scientific panel on contaminants in food chain on a request from the commission related to fumonisins as undesirable substances in animal feed. EFSA J (2005) 3(7):1–32. 10.2903/j.efsa.2005.235

[20] Bognanno M La Fauci L Ritieni A Tafuri A De Lorenzo A Micari P Survey of the occurrence of Aflatoxin M1 in ovine milk by HPLC and its confirmation by MS. Mol Nutr Food Res (2006) 50(3):300–5. 10.1002/mnfr.200500224 16523445

[21] Partanen HA El-Nezami HS Leppänen JM Myllynen PK Woodhouse HJ Vähäkangas KH. Aflatoxin B1 transfer and metabolism in human placenta. Toxicol Sci (2010) 113(1):216–25. 10.1093/toxsci/kfp257 19875679

[22] Bodine AB Luer CA Gangjee SA Walsh CJ. In vitro metabolism of the pro-carcinogen aflatoxin B1 by liver preparations of the calf, nurse shark and clearnose skate. Comp Biochem Physiol C (1989) 94(2):447–539. 10.1016/0742-8413(89)90096-0 2576780

[23] Micco C Miraglia M Onori R Brera C Mantovani A Ioppolo A Long-term administration of low doses of mycotoxins to poultry. 1. Residues of aflatoxin B1 and its metabolites in broilers and laying hens. Food Addit Contam (1988) 5(3):303–8. 10.1080/02652038809373708 3135210

[24] Trucksess MW Richard JL Stoloff L McDonald JS Brumley WC. Absorption and distribution patterns of aflatoxicol and aflatoxins B1 and M1 in blood and milk of cows given aflatoxin B1. Am J Vet Res (1983) 44(9):1753–6. 6414350

[25] Helferich WG Baldwin RL Hsieh DP. [14C]-aflatoxin B1 metabolism in lactating goats and rats. J Anim Sci (1986) 62(3):697–705. 10.2527/jas1986.623697x 3084435

[26] Carvajal M Rojo F Méndez I Bolaños A Aflatoxin B1 and its interconverting metabolite aflatoxicol in milk: the situation in Mexico. Food Addit Contam (2003) 20(11):1077–86. 10.1080/02652030310001594478 14668158

[27] Carvajal M Bolaños A Rojo F Méndez I Aflatoxin M1 in pasteurized and ultrapasteurized milk with different fat content in Mexico. J Food Prot (2003) 66(10):1885–92. 10.4315/0362-028X-66.10.1885 14572228

[28] Blankenship LT Dickey JF Bodine AB. In vitro mycotoxin binding to bovine uterine steroid hormone receptors. Theriogenology (1982) 17(3):325–31. 10.1016/0093-691X(82)90092-9 16725693

[29] FAO. Forestry Department, Global Forestry Resources Assessment. (2010).

[30] Goyal RK Prevention and control of mycotoxins in foodgrains in India. In: Semple RL Frio AS Hicks PA Lozare JV, editors. Mycotoxin Prevention and Control in Food Grains. Bankok: UNDP/FAO Regional Network Inter-Country Cooperation on Postharvest Technology and Quality Control of Foodgrains, ASEAN Grain Post-Harvest Programme. AGPP Publication (1991).

[31] EFSA. Report on stakeholders’ activities in the area of emerging risks. EFSA J (2011) 8(6):1–40. 10.2903/sp.efsa.2011.EN-170

[32] EFSA. Identification of emerging risks: an appraisal of the procedure trialled by EFSA and the way forward. EFSA J (2015) 12(6):1–30. 10.2903/sp.efsa.2015.EN-824.

[33] European Commission. Regulation (EC) No 178/2002 of the European Parliament and of the Council of 28 January 2002 laying down the general principles and requirements of food law, establishing the European Food Safety Authority and laying down procedures in matters of food safety. Off J. (2002) L031:P0001–24.

[34] IARC. Risk assessment and risk management of mycotoxins. IARC Sci Publ (2012) 158:105–17. 23477199

[35] Monda EO Alakonya AE A review of agricultural aflatoxin management strategies and emerging innovations in sub-Saharan Africa. Afr J Food Agric Nutr Dev (2016) 16(3):11126–38. 10.18697/ajfand.75.ILRI11

[36] Aragon YA Rodrigues I Hofstetter U Binder EM Mycotoxins in silages: occurrence and prevention. Iran J Appl Anim Sci (2011) 1(1):1–10.

[37] Pettersson H Holmberg T Larsson K Kaspersson A Aflatoxins in acid-treated grain in Sweden and occurrence of aflatoxin M1 in milk. J Sci Food Agric (1989) 48:411–20. 10.1002/jsfa.2740480403

[38] Wambacq E Vanhoutte I Audenaert K De Gelder L Haesaert G. Occurrence, prevention and remediation of toxigenic fungi and mycotoxins in silage: a review. J Sci Food Agric (2016) 96(7):2284–302. 10.1002/jsfa.7565 26676761

[39] Boudergue C Burel C Dragacci S Favrot MC Fremy JM Massimi C Review of mycotoxin-detoxifying agents used as feed additives: mode of action, efficacy and feed/food safety. EFSA J (2009) 6(9):1–192. 10.2903/sp.efsa.2009.EN-22

[40] CEC European Commission. White Paper on Food Safety. COM (1999) 719 Final. Brussels (2000). Available from: http://ec.europa.eu/dgs/health_food-safety/library/pub/pub06_en.pdf

[41] Nyamete FA Potential of lactic acid fermentation in reducing aflatoxin B1 and fumonisin B1 in Tanzanian maize-based complementary gruel. Afr J Food Agric Nutr Dev (2016) 16(3):11139–151. 10.18697/ajfand.75.ILRI12

[42] Megalla SE Mohran MA. Fate of aflatoxin B-1 in fermented dairy products. Mycopathologia (1984) 88(1):27–9. 10.1007/BF00439291 6440020

[43] European Commission. Commission regulation (EC) No 1881/2006 of 19 December 2006 setting maximum levels for certain contaminants in foodstuffs. Official J Eur Union (2006) L 364:5–24.

[44] European Commission. Regulation (EC) No 1069/2009 of the European Parliament and of the Council of 21 October 2009 laying down health rules as regards animal by-products and derived products not intended for human consumption and repealing regulation (EC) No 1774/2002 (Animal by-products regulation). Official J Eur Union (2009) 300:1–33.

[45] FSSAI. Food Safety and Standards (Contaminants, Toxins and Residues) Regulations. (2011).

[46] Ministero della Salute. Aflatoxins contamination in Corn and Food Chain. Italy: Dept. Veterinary Public Health and Food Safety (2013).

[47] CEC. Council Directive 96/23/EC of 29 April 1996 on measures to monitor certain substances and residues thereof in live animals and animal products and repealing Directives 85/358/EEC and 86/469/EEC and Decisions 89/187/EEC and 91/664/EEC. Off J (1996) L125:10–32.

[48] Warth B Sulyok M Krska R. LC-MS/MS-based multibiomarker approaches for the assessment of human exposure to mycotoxins. Anal Bioanal Chem (2013) 405:5687–95. 10.1007/s00216-013-7011-1 23774829 PMC3695324

[49] Frazzoli C Mantovani A Dragone R Local role of food producers’ communities for a Global One-Health framework: the experience of translational research in an Italian dairy chain. J Agric Chem Environ (2014) 3(2B):14–9. 10.4236/jacen.2014.32B003

[50] Frazzoli C Bocca B Mantovani A The one health perspective in trace elements biomonitoring. J Toxicol Environ Health B Crit Rev (2015) 18(7–8):344–70. 10.1080/10937404.2015.1085473 26691900

[51] Cheng R Mantovani A Frazzoli C Analysis of food safety and security challenges in emerging African food producing areas through a One Health lens: the dairy chains in Mali. J Food Prot (2017) 80(1):57–67. 10.4315/0362-028X.JFP-15-561 28221872

[52] Dragone R Ermilov L Grasso G Maggioni S Mantovani A Frazzoli C. Antioxidant power as biochemical endpoint in bread for screening and early managing quality and toxicant-related safety anomalies in food production. Food Chem Toxicol (2016) 94:31–8. 10.1016/j.fct.2016.04.028 27174639

[53] Dragone R Frazzoli C Grappelli C Campanella L. A new respirometric endpoint-based biosensor to assess the relative toxicity of chemicals on immobilized human cells. Ecotoxicol Environ Saf (2009) 72:273–9. 10.1016/j.ecoenv.2008.02.011 18499252

[54] Legator M Biological effects of aflatoxin in cell culture. Bacteriol Rev (1966) 30(2):471–7. 5327761 10.1128/br.30.2.471-477.1966 PMC441007

[55] Sergeev IN Arkhapchev luP Kravchenko LV Kodentsova VM Piliia NM Effect of mycotoxins aflatoxin B1 and T-2 toxin on the vitamin D3 metabolism and binding of its hormonal form 1,25-dihydroxyvitamin D3 in rats. [Article in Russian]. Vopr Med Khim (1988) 34(4):51–7. 2848363

[56] Flores-Flores ME Lizarraga E Lopez de Cerain A Gonzalez-Penas E Presence of mycotoxins in animal milk: a review. Food Control (2015) 53:163–76. 10.1016/j.foodcont.2015.01.020

